# Simultaneous Treatment of Both Sides of the Polymer with a Conical-Shaped Atmospheric Pressure Plasma Jet

**DOI:** 10.3390/polym15020461

**Published:** 2023-01-16

**Authors:** Felipe Vicente de Paula Kodaira, Bruno Henrique Silva Leal, Thayna Fernandes Tavares, Antje Quade, Luis Rogerio de Oliveira Hein, William Chiappim, Konstantin Georgiev Kostov

**Affiliations:** 1 Laboratory of Plasmas and Applications, Department of Physics, Faculty of Engineering and Sciences, São Paulo State University (UNESP), Guaratinguetá, São Paulo 12516-410, Brazil; 2 Leibniz Institute for Plasma Science and Technology—INP, 17489 Greifswald, Germany; 3 Department of Materials and Technology, São Paulo State University, UNESP, Guaratinguetá, São Paulo 12516-410, Brazil

**Keywords:** conical-shaped, atmospheric pressure plasma jet, polymer, polypropylene, SEM, FTIR, XPS

## Abstract

A conical-shaped atmospheric pressure plasma jet (CS-APPJ) was developed to overcome a standard limitation of APPJs, which is their small treatment area. The CS-APPJs increase the treatment area but use the same gas flow. In the present work, polypropylene samples were treated by CS-APPJ and characterized by scanning electron microscope (SEM), the contact angle, Fourier-transformed infrared spectroscopy (FTIR), and X-ray photoelectron spectroscopy (XPS). It was observed that the treatment co-occurs on the face directly in contact with the plasma and on the opposite face (OF) of the samples, i.e., no contact. However, the treatment changed the chemical composition on each side; the OF is rougher than the direct contact face (DCF), probably due to the oxygen groups in excess at the DCF and nitrogen in quantity at the OF. Although simultaneous treatment of both sides of the sample occurs for most atmospheric plasma treatments, this phenomenon is not explored in the literature.

## 1. Introduction

Although surface modification of polymers by non-thermal atmospheric pressure plasmas (NTAPPs) has become a well-established technology [1,2,3], there is still much to be improved in this device development and optimization. The processing of materials by NTAPP has several advantages over other industrial processes, highlighting the advantage of being eco-friendly, i.e., it does not involve potentially dangerous solvents to the environment or workers [4,5]. Its versatility in modifying the surface of polymers without changing the bulk properties is worth mentioning, in addition to being suitable for most polymers that are sensitive to high temperatures [6,7,8]. Furthermore, plasma processes can take place in an open or controlled environment, with the treatment time varying from a few seconds to several minutes, depending on the configuration of the device used [8,9,10,11,12]. It is worth mentioning that this process can be carried out continuously in a line of production or individually [13,14]. Among the NTAPP devices, dielectric barrier discharge (DBD) is the most widely used [11,12,13,14,15,16,17,18,19,20]. A significant advantage of DBD over other devices that generate electrical discharges is the higher electron density induced by micro-discharges caused by a large number of tiny current filaments that pass through the dielectric material covering one or both electrodes [21,22]. This high electron density improves the functionalization of the polymers due to the uniform incidence of discharge on the treated surface [6,8]. However, the maximum distance between the electrodes is limited, affecting the device’s operation in open systems [23].

In the late 1990s, atmospheric pressure plasma jets (APPJs) appeared to mitigate, reduce or suppress the deficiency mentioned above. APPJs generate plasma plumes in open spaces, enabling the direct treatment of samples of different shapes and sizes [24,25,26,27,28,29,30,31,32,33,34]. Over the years, APPJs have been successfully tested in many applications, such as material treatment, sterilization, cancer and wound treatment, aesthetic applications, and dentistry [35,36,37,38,39,40,41,42,43,44,45,46,47,48,49]. Many different configurations of APPJs are reported in the literature. In his review article Lu, X et al. [32] presented APPJ with a single electrode, APPJ without dielectrics, DBD jets with ring and pin electrodes, and combinations between them.

However, plasma jets have diameters of a few millimeters, making them suitable for biomedical applications [24,29,30,31,50]. It is known that the most intense effect of APPJ is in the central region, with a gradual decrease as it moves away from the plasma plume region. Thus, its minor diameter limits the treatment area or surface activation, which makes its application time-consuming and expensive for industrial applications. The exposure time to APPJ can be increased to overcome the technological limitation, which increases the treatment area or surface activation [51,52]. The problem with increasing exposure time to APPJ is that there may be damage to the treated surface. Therefore, there is a need to improve the APPJs for use in larger areas. Recently, Abdelaziz et al. [53] investigated a configuration of a wide tube APPJ with a diameter of up to 30.0 mm. However, for this configuration, the gas flow used is high, which generates a non-uniform movement of the gas inside wide tubes [53]. Some works have tried to enlarge the diameter of the APPJs using complex and 2D jet arrays [54,55]. However, these configurations bring challenges, such as interactions between neighboring jets [56,57] and increased gas consumption, which increases with the number of jets in the array. Another exciting configuration is a gas-flowless pin-to-ring geometry presented by Khun et al. [58], where they reported achieving a more extensive treatment area than most APPJs.

In order to improve and explore novel APPJ configurations, this work used a conical-shaped APPJ (CS-APPJ) with a 75.0 mm diameter outlet nozzle. Discharge was initiated between a high voltage pin electrode inserted into the thin part of the conical-shaped (CS). In contrast, a grounded flat electrode covered by a 4.0 mm thick glass insulator was inserted under the CS. The glass plate also served as a sample holder. Compared to most APPJs, this device allows treatment over a significantly larger area, allowing for greater sample thickness and shape variety. The surface modification effect extends over the entire area covered by the CS and exhibits a considerable degree of uniformity.

Furthermore, our results prove that plasma treatments co-occur on both sides of the flat samples. Although the discharge geometry resembles a point-to-plane corona configuration, it also has a dielectric barrier covering the ground electrode, which makes it a DBD-type device. Finally, due to the gas flow required for the discharge propagation, the system also has some similarities with the APPJs. However, in the case of the CS-APPJ, there is no plasma plume but rather a filament discharge composed of multiple filaments that start from the top electrode pin and travel along the inner wall of the funnel to end in the glass substrate. The polymer chosen for this work was polypropylene (PP) because it is a common material widely used in several industrial applications, such as packaging, decoration, electronics, and medicine [59]. The extensive use of PP comes from its versatility, stability, and good mechanical properties [59]. However, as with most polymeric materials, PP has low surface energy, making it difficult to paint or glue [60,61]. Plasma processing of polymers conveniently increases their surface energy while keeping the beneficial bulk properties unchanged [60,61].

Therefore, the present work contributes to developing plasma jets, mainly of the DBD type. One can also highlight the contribution in the application of the device on polymeric surfaces, which are of great applicability in industry and the daily life of the entire world population.

## 2. Materials and Methods

### 2.1. Assembly Setup and Electrical Measurements

**Figure 1:**
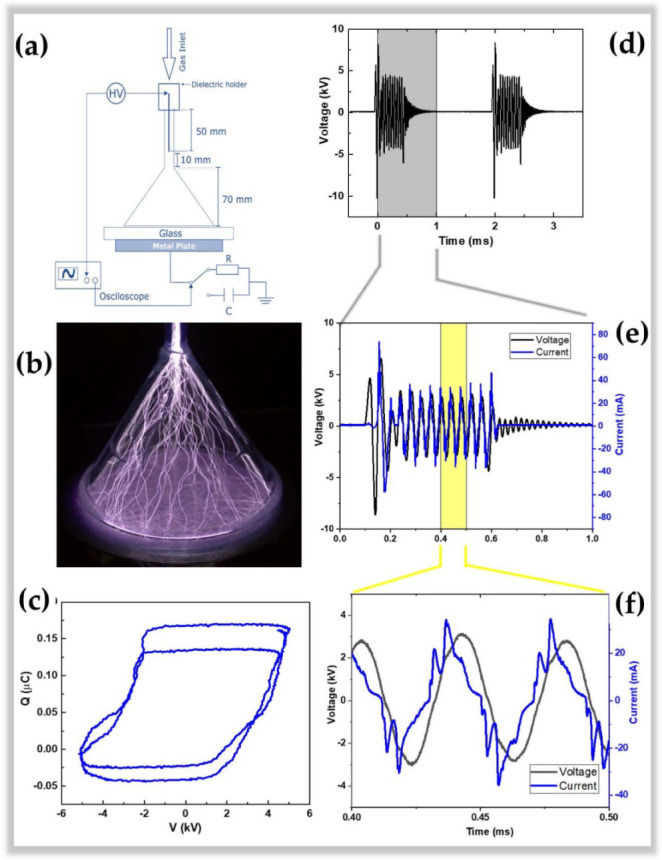
(**a**) Schematic layout of the conical-shaped atmospheric pressure plasma jet (CS-APPJ) system; (**b**) Photo of the filamentary discharge inside the conical horn; (**c**) Lissajous Figure (Q × V). Overview of typical electrical parameters of (**d**) applied voltage; (**e**) voltage and current waveforms in a full burst of 12 high-voltage oscillations; (**f**) in two wave periods from the middle of the burst signal.

The device employs a 3.0 mm thick commercial glass funnel (conical-shaped) comprised of an 8.0 mm narrow tube section ending in a 75.0 mm conical horn. It is placed vertically with its wide part facing downwards (see Figure 1a), while the top of the funnel is closed by a dielectric support through which a 1.0 mm diameter pin electrode is introduced. The pin-to-plate electrode geometry is formed by placing a grounded plate electrode under the 4.0 mm thick glass-covered funnel, which also serves as a sample holder. Working gas (99.2% Ar from AirLiquid, São José dos Campos, Brazil) was injected into the system through an orifice in the dielectric support at a flow rate of 2.0 SLM. The sharp pin electrode was connected to a commercial high-voltage power supply (model Minipuls 6, GBS Elektronik GmbH, Radeberg, Germany). It was operated in burst mode, i.e., generating 12 consecutive high voltage oscillations at a frequency of 25.0 kHz followed by a period of voltage off. The burst repetition period was set to 2.0 ms. Electrical characterization was performed by obtaining the applied voltage (directly from a voltage divider in the power supply) and calculating the transferred charge and discharge current by measuring the voltage drop across a serial capacitor (10.0 nF) or resistor (120.0 Ω), respectively, coupled as shown in Figure 1a. The discharge power was calculated from the electrical energy contained in a burst (calculated by the area of the Q-V Lissajous figure) divided by the repetition period [62]. A photo of the filament discharge generated inside the funnel with 2.0 SLM Ar is shown in Figure 1b. As can be seen, the discharge filaments start at the tip of the high voltage electrodes, and after reaching the glass funnel, the inner wall propagates downstream along the conical horn until it reaches the glass substrate. The discharge power was calculated as 8.0 ± 0.2 W, using the area of the Q × V Lissajous figure of an entire burst period. Figure 1c shows the Lissajous figure of a cutout of two oscillations within the burst. Figure 1d depicts the typical voltage signal showing two consecutive (high-voltage) HV bursts of 12.0 cycles with a 2.0 ms repetition period. In contrast, a detailed view of the voltage and current waveforms in a burst is shown in Figure 1e. For this, a magnification interval between 0.0 and 1.0 ms was used. Finally, Figure 1f shows a clearer view of the signal indicating only two periods of oscillation (magnification between 0.40 and 0.50 s). The current signal in Figure 1f exhibits the typical form of a DBD discharge, i.e., multiple current spikes superimposed on the capacitive current. Thus, although the geometry of the discharge resembles a point-to-plane corona configuration, the dielectric barrier that covers the ground electrode makes it a DBD-type device.

Therefore, this new CS-APPJ configuration used in this work can be characterized as a new DBD-type APPJ and is an essential contribution to the field of plasma surface treatment. It is important to note that in the case of CS-APPJ, there is no plasma plume but a filament discharge composed of several filaments that start at the pin of the upper electrode and run along the inner wall of the funnel, and end at the substrate (as can be seen in Figure 1b).

### 2.2. Polypropylene Samples Preparation

The polypropylene flat samples (0.95 g/cm^3^; 1.0 mm thickness) were cut into rectangular shapes (20.0 × 10.0 mm^2^), then cleaned for 20.0 min in an ultrasonic bath with distilled water and finally rinsed in isopropanol for another 20.0 min. Finally, the samples were dried at room temperature and treated up to 5.0 min. 

### 2.3. Characterization of Polypropylene Samples

Scanning electron microscope (SEM) images were performed by a Carl Zeiss EVO LS 15 tool at low pressure (~10^−3^ Pa) and high electron tension of 5.0 kV. Before the measurements, 20.0 × 10.0 mm samples were covered by a 6.0 nm thick gold layer deposited by magnetron sputtering. Topography images were carried out from both the direct contact face (DCF) and opposite face (OF) of samples. In addition, the roughness values were obtained from the SEM images. The wettability was carried out on a Ramé-Hart 300 F1 goniometer by depositing deionized water droplets (1.0 µL). The droplets were placed along the longer sample axis spaced 5.0 mm from each other, and nine samples were measured for each treatment time. Both faces were measured. The mean water contact angle (WCA) value of the droplets on all samples was then calculated and plotted to evaluate the dependence on the treatment time and the difference of the treatment on both faces of the PP samples. The changes in the molecular structure of PP samples were carried out by Fourier-transform infrared spectroscopy (FTIR) coupled with attenuated total reflectance (ATR), which was obtained by a Perkin Elmer Spectrum 100 FTIR spectrometer. X-ray photoelectron spectroscopy (XPS) analysis was performed in a Kratos AXIS Ultra. The composition of the surface was scanned along its longest symmetry axis, looking for carbon, oxygen, and nitrogen. Again, both faces of the samples were analyzed. XPS data were also used to ascertain the homogeneity of the treatment by inspecting the chemical composition along the surface of the samples.

## 3. Results and Discussion

The results obtained were discussed separately for each characterization technique in this section. In the SEM images, it is possible to observe differences in both faces of the sample, with the formation of oligomers of low molecular weight oxidized materials (LMWOM) in the OF. The constant water angle measurements corroborate the hypothesis that the structures formed on the underside are LMWOM; as these groups are polar, lower contact angle values are expected [18], as shown in Section 3.2. The spectroscopic characterization techniques (FTIR and XPS) confirm different structures and chemical compositions for both sides of the samples treated with oxygen groups in DFC and nitrogen groups in OF.

### 3.1. Scanning Electron Microscopy (SEM)

**Figure 2:**
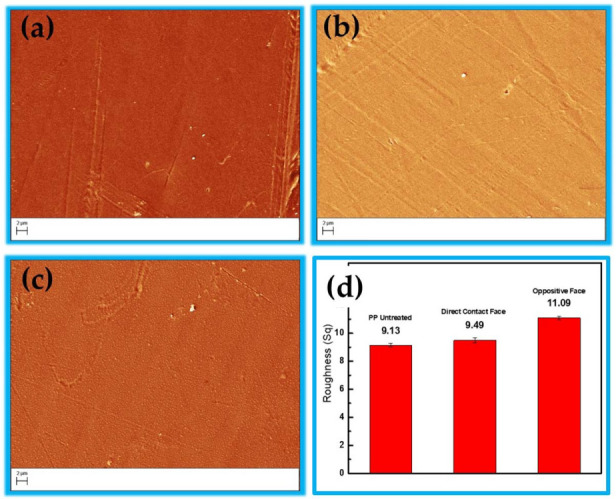
Scanning electron microscopy (SEM) images of polypoprylene samples: (**a**) untreated; (**b**) treated by plasma with direct face contact (DFC); (**c**) treated by plasma through the opposite face (OF); and (**d**) roughness values for all samples. All samples were treated for 3 min.

Figure 2 shows SEM images at 5000× *g* magnification. Figure 2a shows the PP without treatment, and Figure 2b,c show the images of the PP with the face directly in contact with the discharge and the opposite face, respectively. Figure 2d shows the roughness of the respective samples, evaluated from the images in Figure 2a,b,c. As can be seen, the untreated surface has less roughness compared to the PP treated for 5.0 min. Another interesting point concerns DCF (Figure 2b), which presents a slightly higher surface roughness than untreated samples. In contrast, Figure 2c shows the OF treated under the same conditions; however, it has a greater surface roughness with some small granular structures that spread over the entire surface. The granular forms are probably oligomers, known as oxidized low molecular weight materials (LMWOM). The formation of this type of structure on the surface of the sample is typically observed in the case of polymers treated with DBD and directly applied APPJs [6,18,35]. It is possible to follow in Figure 1b that the discharge filaments run along the wall of the conical structure of the reactor and on the surface of the sample holder during the treatment. However, in polymer treatment, the filaments also pass under the sample, which makes the bottom side of the sample closer to the discharge. In addition, due to the loading of charges on the sample surface due to the sample/plasma contact, there is the generation of an electric field between the sample surface and the sample holder, which can lead to the formation of a DBD-planar type under the bottom side of the sample. This generation of a DBD discharge in the OF may be the cause of the greater roughness in this face compared to the DFC.

Another point that may contribute to the different roughness of the OF is the lower pressure of the working gas (Ar) that reaches the surface of the polymer. In contrast, there is a more significant interaction with free radicals from the formation of the planar type DBD generated between the sample surface and the sample holder, which increases the energy of electrons and free radicals, increasing ablation and interactions with reactive molecules such as N_2_+, N_4_+, N+, O_2_+, H_2_O+, O_2_− and O− [8,63]. However, due to the high complexity of the interaction between the plasma and the polymer surface, it is not possible to differentiate by SEM images whether the attack is related to the reduction of the chemical structure of the surface caused by plasma or is associated with the material removed through the impact of surface plasma species [8,63].

### 3.2. Water Contact Angle (WCA)

**Figure 3:**
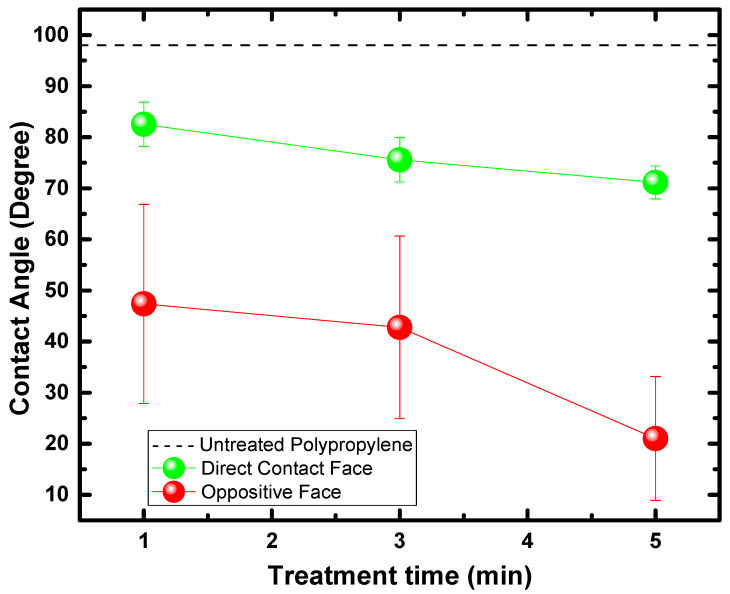
Mean values of water contact angle of polypropylene samples measured on DCF with plasma and OF for 1.0, 3.0, and 5.0 min of treatment.

On both sides, the contact angle with water (WCA) was measured on 20.0 × 10.0 mm PP samples. The WCA of the untreated substrate was 97.1°, according to [64,65]. Mean values are shown in Figure 3, these values were measured along the longest axis of the sample, spaced 5.0 mm apart, and nine samples were measured for each treatment time, thus obtaining an average value with an error bar (as seen in Figure 3). On both sides, the treatment promoted a reduction in the WCA. It can be observed that the treatment of DFC presented WCA between 70 and 80 degrees.

In contrast, the OF showed a more pronounced WCA reduction, with values below 50 degrees. Another point to be observed is the error bars that present variations of up to 20 degrees concerning the average value, which indicates a less homogeneous treatment in the OF about the DCF. According to Morent et al. [66], the considerable reduction in WCA of PP treated with DBD is due to the formation of functionalities containing oxygen, with a prevalence of C–O, O–C=O, and C=O. Other authors show similar results for other polymeric substrates, such as PA6 and PA66 [67]. Therefore, this behavior corroborates the hypothesis suggested by the SEM images, which assumes the formation of LMWOM in the substrate treated with predominance in the OF, which favors the reduction of WCA.

### 3.3. Chemical Analysis of the Treated and Untreated Polypropylene

**Figure 4:**
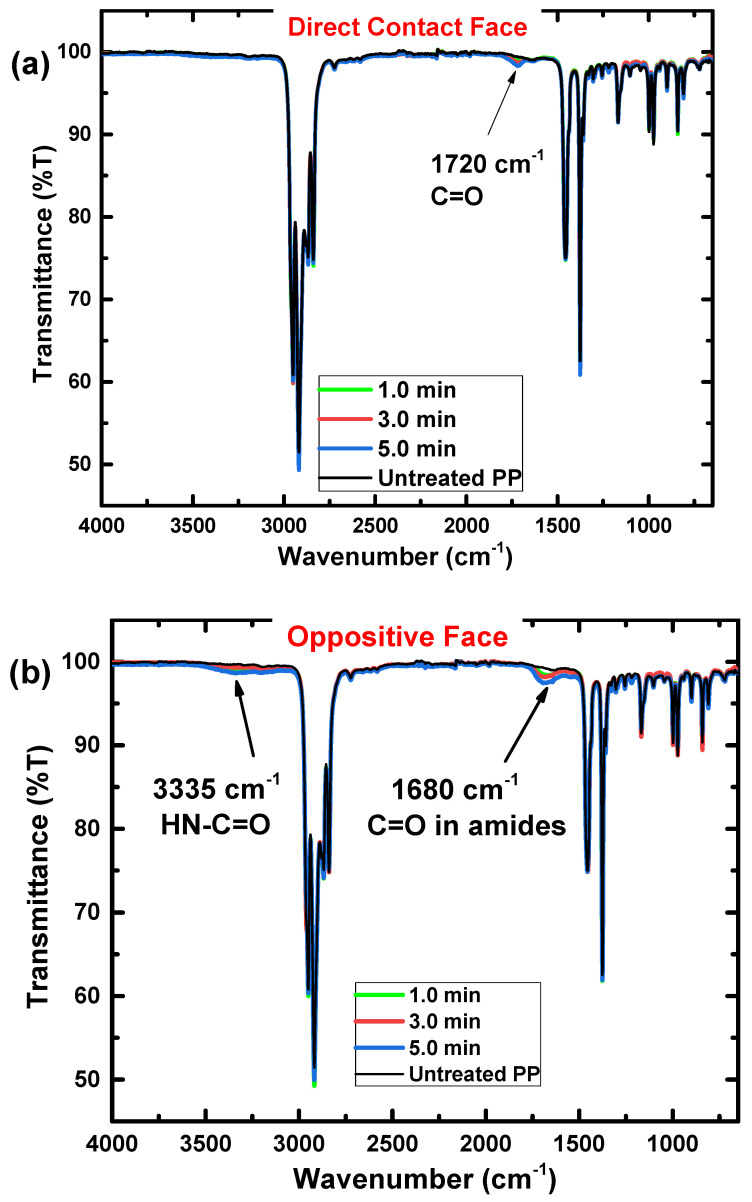
FTIR full spectra of polypropylene samples untreated, and treated by 1.0, 3.0, and 5.0 min. (**a**) DCF; and (**b**) OF.

Figure 4 shows the FTIR spectra for PP samples treated with CS-APPJ for periods of 1.0, 3.0, and 5.0 min, in addition to the spectrum of the untreated PP sample. It is essential to highlight that due to the reach of the technique, which varies between 0.5–5.0 µm (4000–400 cm^−1^) in depth, FTIR becomes an excellent tool for understanding and verifying possible chemical changes in the inner layers of the polypropylene. Figure 4a shows the bands related to the DCF internal connections. In contrast, Figure 4b shows the OF-related bands. 

The absorption bands associated with PP demonstrated in Figure 4a,b are (i) bands related to the asymmetric elongation vibration –CH_3_ at 2952 cm^−1^; (ii) bands related to symmetric flexion –CH_2_–, symmetric elongation –CH_2_– and asymmetric elongation –CH_2_–, respectively, at 1455, 2838 and 2917 cm^−1^; (iii) symmetric bending vibration bands of the –CH_3_ group is detected at 1375 cm^−1^; (iv) the bands attributed to the oscillating vibration –CH_3_ are at 972, 997 and 1165 cm^−1^; (v) the band located at 840 cm^−1^ is attributed to the C-CH_3_ stretching vibration. It is essential to highlight that the bands mentioned above appear due to the presence of the methyl group in polypropylene [66,67,68,69,70]. After CS-APPJ treatment for periods of 1.0, 3.0, and 5.0 min, new bands were detected in the FTIR spectra. First, in DCF (Figure 4a), a peak appears at 1720 cm^−1^ related to C=O groups [23]. Its intensity increases with treatment time. In contrast, Figure 4b shows that in the OF of the sample, a new band at approximately 1680 cm^−1^ is observed. This new band is only detected in the OF of the treated samples and can be attributed to C=O stretching groups in amides. Figure 4b also shows a band at approximately 3335 cm^−1^, which may be associated with elongation in the HN-C=O group [71], confirming the presence of nitrogen detected in the 1680 cm^−1^ band.

**Figure 5:**
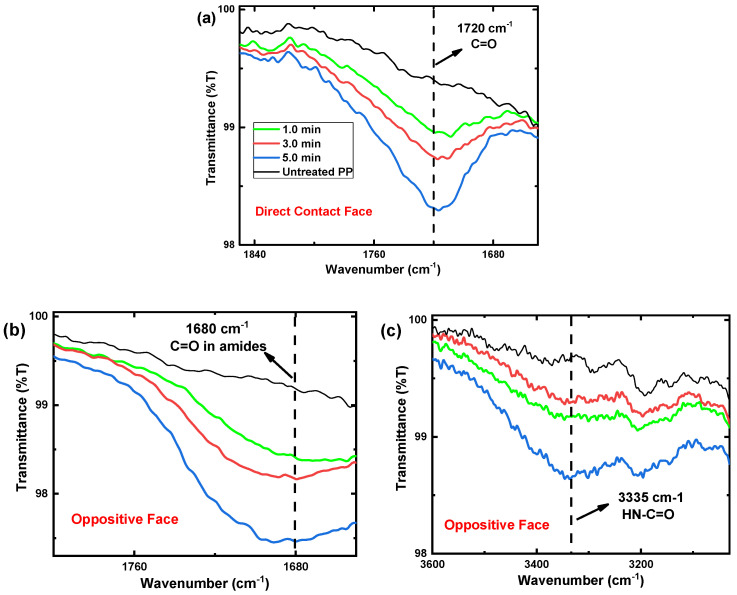
FTIR spectra of polypropylene samples untreated, and treated by 1.0, 3.0, and 5.0 min. (**a**) DCF in the region between 1850 to 1650 cm^−1^; (**b**) OF in the region between 1800 to 1650 cm^−1^; and (**c**) OF in the region between 3600 to 3000 cm^−1^.

Figure 5 shows the growth in intensity of bands associated with CS-APPJ treatment (1720, 1680, and 3335 cm^−1^). In DCF (Figure 5a), it is observed that the C=O groups grows in intensity with the increase in the period of exposure to CS-APPJ. The same behavior is kept for the C=O stretching groups in amides and elongation in the HN-C=O group, referring to OF (Figure 5b).

**Figure 6:**
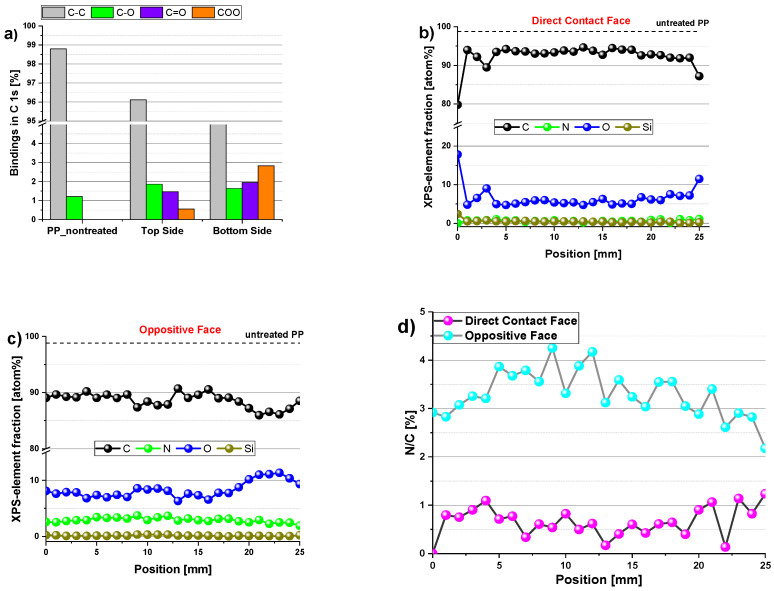
Distribution of the elemental composition of the plasma-treated PP samples; (**a**) Comparison of different carbon groups detected on the untreated PP and both sides of the plasma-treated sample; (**b**) on the DCF; (**c**) on the OF; (**d**) longitudinal distribution of the N/C ratio on both sample sides.

FTIR spectra show that plasma treatment on polymers often results in incorporating oxygen and nitrogen atoms into the material’s surface [72]. Therefore, to study the degree of chemical modifications on the surface of substrates with a depth range between 5.0 and 10.0 nm, XPS analysis was used (Figure 6). With this technique, it is possible to evaluate the surface oxidation and the percentage of atomic species on the surface of the PP. For this, the sum of C, O, N, and Si was considered 100%, i.e., the proportion of H in the calculations was neglected. Figure 6a shows the different groups of carbon bonds detected in the untreated and the samples treated for 5.0 min, and it can be observed that the formation of other groups of carbon bonds occurs in the DCF and the OF, compared with the PP untreatment. 

This corroborates the data found in the FTIR spectra. The untreated sample was observed to contain carbon primarily with only traces of O (less than 2%) due to surface oxidation. After treatment with CS-APPJ, the amount of O on both sides of the sample increased. The OF presented a higher oxygen content with improved COO groups (Figure 6a). This result corroborates the WCA data (Figure 3), which shows a marked reduction of the WCA in the OF. To assess the distribution of chemical elements on both sides of the PP sample treated for 5.0 min, a piece of PP of area 25.0 × 20.0 mm^2^ was used for high-resolution XPS scan measurements along the central axis (25.0 mm). The results are shown in Figure 6b,c, indicating that the CS-APPJ treatment added O atoms and a small amount of N with only traces of Si atoms on the PP surface. Figure 6b,c show that the species are homogeneously distributed along both sides of the sample. This shows the uniformity in the treatment with CS-APPJ, which corroborates the SEM images (Figure 2). Figure 6d shows the N/C percentage ratio of both faces, where a balance between 0.0 and 1.0% (DCF) and between 2.5 and 4.0% is observed in the phase opposite to direct contact with the plasma. It is essential to highlight that the XPS results corroborate the FTIR spectra, showing the formation of different groups on opposite sides of the treated samples.

## 4. Conclusions

Despite resembling the geometry of a point-to-plane corona configuration, the CS-APPJ device behaves similar to a DBD-type device, as observed in the typical electric current signal shown in Figure 1. In the CS-APPJ, the filaments of discharge run along the wall of the conical structure until they reach the surface of the sample holder during the treatment. The formation of a DBD-planar under the OF of the sample was observed due to the loading of charges on the sample surface caused by the sample/plasma contact. The generation of a planar-DBD under the OF was responsible for simultaneously treating both sides of the PP, which led to different surface changes in the OF compared to the DCF. Treatment with the CS-APPJ device induced physical and chemical changes on the surface of commercial PP. The SEM images show slight surface alterations, with a more significant modification occurring in the OF samples that present greater roughness. Corroborating the SEM and roughness images, the WCA measurements show the formation of LMWOM, which favors the reduction of WCA with increasing treatment time with CS-APPJ. The FTIR analysis showed that the treatment with CS-APPJ affected the internal bonds in the deep layer of the polymer in different ways, with the detection of the presence of amide groups in the OF and C=O in the DCF. Furthermore, FTIR spectra indicated that the amount of these functional groups tends to increase with treatment time. The XPS measurements showed uniformity in the surface treatment on both sides of the sample, highlighting the difference in functional groups incorporated on both sides of the material, with a more significant presence of nitrogen groups in the OF, corroborating the FTIR spectra.

## Data Availability

Not applicable.
